# Assessment of Bone Mineral Status in Children With Marfan Syndrome

**DOI:** 10.1002/ajmg.a.35540

**Published:** 2012-08-07

**Authors:** Monica Grover, Nicola Brunetti-Pierri, John Belmont, Kelly Phan, Alyssa Tran, Roman J Shypailo, Kenneth J Ellis, Brendan H Lee

**Affiliations:** 1 Department of Pediatric Diabetes and Endocrinology, Baylor College of MedicineHouston, Texas; 2 Department of Molecular and Human Genetics, Baylor College of MedicineHouston, Texas; 3 Children's Nutrition Research Center, Baylor College of MedicineHouston, Texas; 4 Howard Hughes Medical Institute, Baylor College of MedicineHouston, Texas

**Keywords:** Marfan syndrome, bone mineral density, TGF-β

## Abstract

Marfan syndrome (MFS) is an autosomal dominant connective tissue disorder with skeletal involvement. It is caused by mutations in fibrillin1 (*FBN1*) gene resulting in activation of TGF-β, which developmentally regulates bone mass and matrix properties. There is no consensus regarding bone mineralization in children with MFS. Using dual-energy X-ray absorptiometry (DXA), we evaluated bone mineralization in 20 children with MFS unselected for bone problems. *z*-Scores were calculated based on age, gender, height, and ethnicity matched controls. Mean whole body bone mineral content (BMC) *z*-score was 0.26 ± 1.42 (*P* = 0.41). Mean bone mineral density (BMD) *z*-score for whole body was −0.34 ± 1.4 (*P* = 0.29) and lumbar spine was reduced at −0.55 ± 1.34 (*P* = 0.017). On further adjusting for stature, which is usually higher in MFS, mean BMC *z*-score was reduced at −0.677 ± 1.37 (*P* = 0.04), mean BMD *z*-score for whole body was −0.82 ± 1.55 (*P* = 0.002) and for lumbar spine was −0.83 ± 1.32 (*P* = 0.001). An increased risk of osteoporosis in MFS is controversial. DXA has limitations in large skeletons because it tends to overestimate BMD and BMC. By adjusting results for height, age, gender, and ethnicity, we found that MFS patients have significantly lower BMC and BMD in whole body and lumbar spine. Evaluation of diet, exercise, vitamin D status, and bone turnover markers will help gain insight into pathogenesis of the reduced bone mass. Further, larger longitudinal studies are required to evaluate the natural history, incidence of fractures, and effects of pharmacological therapy. © 2012 Wiley Periodicals, Inc.

## INTRODUCTION

Marfan syndrome (MFS) is an autosomal dominant connective tissue disorder with variable expressivity and skeletal, cardiovascular, and ocular involvement. The estimated prevalence is about 1 per 10,000 persons [Gray et al., 1994]. It is caused by mutations in *FBN1* gene [Dietz et al., 1991; Lee et al., 1991; Maslen et al., 1991] encoding for fibrillin 1, a glycoprotein that aggregate to form microfibrils. It was believed that abnormal microfibrils altered the entire fibril structure (a dominant negative effect) and hence resulted in the disease phenotype. However, certain features like long bone and rib overgrowth and muscle wasting cannot be explained by this phenomenon.

Judge et al. [2004] proposed that *FBN1* haploinsufficiency is a major pathogenetic mechanism of MFS. Fibrillin 1 in microfibrils interacts with the large latent complex (LLC), which consists of latent TGF-β, two precursor peptides and LTBP-1. TGF-β is activated and released from this complex under influence of environmental and molecular signals.

Dietz et al. proposed that the loss of fibrillin 1 protein has a subsequent effect on the pool of TGF-β that via related signaling might give rise to various phenotypic manifestations of the disease. This effect could further be modulated by different variants of *FBN-1* and hence, explain the variability in clinical presentation [Byers, 2004; Judge et al., 2004].

TGF-β is known to positively regulate osteoblast proliferation and differentiation in vitro [Mundy and Bonewald, 1990]. In vivo, TGF- β has been shown to developmentally regulate bone mass and matrix properties as evidenced by low bone mass and poor bone quality in TGF-β^−/−^ mice [Mohammad et al., 2009]. In addition, TGF-β released during bone resorption induces migration of bone mesenchymal stem cells, which further differentiate into osteoblasts. Hence, TGF-β plays a critical role, as a coupling factor of osteoblast and osteoclasts, in bone remodeling in adult mice [Tang et al., 2009]. On the other hand, it is clear that too much TGF-β signaling can also be detrimental. For example, over-expression of TGF-β from osteoblasts leads to low bone mass due to stimulation of osteoclastogenesis [Mohammad et al., 2009]. Because alteration of TGF-β signaling and its pharmacological modulation is critical in the pathogenesis of the vascular disease of MFS [Holm et al., 2011], we hypothesized that this altered TGF-β signaling may also affect the skeleton of MFS patients.

Currently available dual-energy X-ray absorptiometry (DXA) technology has limitations in evaluating bone mineral density and content in large skeletons because it tends to overestimate standardized scores normalized only for age. Several authors have observed reduced axial and peripheral bone mineral density (BMD) in adults (males and/or females) with MFS suggesting that these patients are at increased risk of fractures [Kohlmeier et al., 1993, 1995; Tobias et al., 1995; Le Parc et al., 1999; Carter et al., 2000; Giampietro et al., 2003, 2007; Moura et al., 2006]. Conversely, some authors did not find evidence of osteopenia [Gray et al., 1993]. But, there is a paucity of data regarding bone mineral status of patients with MFS during childhood, an important period of life because inadequate acquisition of bone mass in childhood increases the lifetime risk of developing osteoporosis. To our knowledge, there is one study including children with MFS (n = 16), which showed low BMD at femoral neck and a trend towards reduced lumbar spine BMD [Kohlmeier et al., 1995]. Another study reported normal BMD in MFS children (n = 21) at lumbar spine and femoral neck [Giampietro et al., 2003]. Hence, it is unclear whether bone mineral density is low in children with MFS.

In this study, we investigated the bone mineral status in children with MFS compared to age, gender, and ethnicity matched controls using DXA. The data were then adjusted for the large skeleton of MFS patients using the prediction models developed by Zemel et al. on >1,500 children enrolled in BMD study and validated using an independent cross-sectional sample of >900 healthy children from the reference data project.

## MATERIALS AND METHODS

The Institutional Review Board for human subject research at Baylor College of Medicine approved this cross-sectional study. The research was prospectively reviewed and approved by a duly constituted ethics committee. Patients, diagnosed with MFS based on Ghent Nosology [Loeys et al., 2010], were recruited from Medical Genetics Clinic at Texas Children's Hospital, Houston, Texas. Informed, written consent was obtained from all parents. Height (±0.5 cm) was measured with a stadiometer, and weight (±0.1 kg) was measured on a standard clinical balance. History of β-blocker intake and fractures suffered were obtained. Subjects taking corticosteroids or any other medications affecting bone metabolism or who had hardware inserted for scoliosis surgery were excluded.

Subjects underwent DXA with a Hologic Delphi-A instrument (Software version 11.2) to measure whole body and lumbar spine BMD and bone mineral content (BMC). We compared the patients against a group of controls matched for age, gender, and ethnic background from the database maintained at the Body Composition Laboratory of the Children's Nutrition Research Center at Baylor College of Medicine [Ellis et al., 2001]. We then used the prediction models to adjust for the tall stature of the patients between ages 7 and 17 years (n = 11) [Zemel et al., 2010]. DXA results are presented as *z*-scores.

We used SPSS 19.0 (SPSS Inc., Chicago, IL) for statistical analysis. Comparison of *z*-score mean with the zero mean reference value was made with the one sample *t*-test. Ninety-fifth percentile confidence intervals were calculated.

## RESULTS

Our cohort consisted of 20 children with mean age of 9.4 ± 4.7 years (age range 3.2–18.5 years). There were 14 males and 6 females with different ethnicities representative of the region and diversity of clinics at Texas Children's Hospital: Caucasian (n = 13), Hispanic (n = 3), and African American (n = 4). As expected, most of the patients (n = 16) were >95th percentile height for age with mean height *z*-score of 2.5 ± 1.1. Seventeen out of 20 patients had a normal BMI with *z*-score within ±2.0. Only one patient reported history of two fractures and 16 patients reported taking β-blocker therapy (propranolol, metoprolol, or atenolol; Table I).

**TABLE I:** Patient Characteristics

Number of patients	20
Males/females	14/6
Mean age (range)	9.4 ± 4.7 years (3.2–18.5)
Ethnicity	Caucasian (n = 13)
	Hispanic (n = 3)
	African American (n = 4)
Mean height	144.7 ± 42.5 cm
Number of fractures/patients	2/1
Number taking β-blockers	16

Age-matched *z*-score does not take into account differences in body size between MFS and healthy children of the same age. Therefore, we compared the patients against a group of controls from the database maintained at the Body Composition Laboratory of the Children's Nutrition Research Center at Baylor College of Medicine to recalculate *z*-score adjusted for age, gender, weight, and ethnicity [Ellis et al., 2001].

**FIG. 1:**
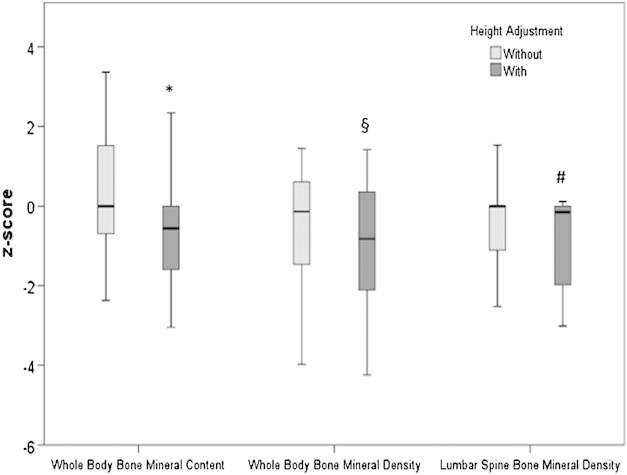
Height adjusted BMC and BMD *z*-scores compared to matched healthy controls. *P = 0.04, ^§^*P* = 0.002, ^#^*P* = 0.001.

We found that mean whole body BMC *z*-score was 0.26 ± 1.42 (*P* = 0.41). Mean BMD *z*-score for whole body was −0.34 ± 1.4 (*P* = 0.29) and for lumbar spine was 0.55 ± 1.34 (*P* = 0.017). On further adjusting for tall stature using recently published prediction models [Zemel et al., 2010], mean whole body BMC *z*-score was statistically reduced at −0.677 ± 1.37 (*P* = 0.04), mean BMD *z*-score for whole body was −0.82 ± 1.55 (*P* = 0.002) and for lumbar spine was −0.83 ± 1.32 (*P* = 0.001; Fig. 1).

## DISCUSSION

The traditional MFS pathogenic theory of dominant negative effect of *FBN-1* mutations has recently been challenged by the haploinsufficiency theory. It has been suggested that loss of fibrillin 1 protein affects the pool of TGF-β, which via related signaling give rise to the disease manifestations.

*Fbn-1*^−/−^ mouse model showed marked dysregulation of TGF-β activation and signaling leading to apoptosis in lung tissue. In addition, perinatally inhibited TGF-β attenuated the apoptosis and reversed lung disease in vivo. This suggests that matrix sequestration of these cytokines is crucial to their regulation [Neptune et al., 2003]. Also, non-canonical TGF-β signaling results in aortic aneurysm in MFS mice [Holm et al., 2011] that was prevented by TGF-β neutralizing antibody or the angiotensin II type 1 receptor (AT1) blocker, losartan [Habashi et al., 2006].

In addition, TGF-β has been shown to developmentally regulate bone mass and matrix properties, as evidenced by low bone mass and poor bone quality of *TGF-β*^*−/−*^ mice [Bondestam et al., 2007; Mohammad et al., 2009]. Pharmacological inhibition of TGF-β type 1 receptor kinase (SD-208) led to both anabolic (increasing osteoblast differentiation and bone formation) and anti-catabolic (decreasing osteoclast differentiation and bone resorption) effects. In vivo, it was shown to improve trabecular bone but interestingly had no effect on cortical bone [Mohammad et al., 2009]. Also, TGF-β antibody (ID11) has been shown to increase BMD, trabecular thickness, and bone volume by increasing osteoblast and reducing osteoclast number [Edwards et al., 2010]. In addition, *Fbn1*^*mgR/mgR*^ mice (model of severe MFS) revealed decreased bone volume and density due to TGF-β driven osteoclastogenesis [Nistala et al., 2010]. These findings support the hypothesis that alteration in the pool of TGF-β leads to alteration in bone mass and matrix properties. Hence, it is plausible that patients with MFS have altered bone mineral content and density.

Since inadequate acquisition of bone in childhood and adolescence leads to increased lifetime risk of osteoporosis and fracture, it is important to recognize whether bone mineral status is affected in children with MFS. In this study, we have shown that these children have significantly lower bone mineral content and density in whole body and lumbar spine as compared to age, sex, height, and ethnicity matched controls. A limitation of our study is that we do not have information regarding calcium intake, exercise, vitamin D status and bone turnover markers that would be helpful to gain insight into etiology. Since in our cohort only one patient had two fractures, it is unclear whether low BMC and BMD in this population will lead to higher incidence of fractures. Also, DXA is a two dimensional analysis and is not an absolute predictor of BMD and/or fracture risk.

The current therapy for MFS includes β-blockers, which slow the rate of progression of the aortic root dilation but do not influence ocular and pulmonary manifestations of the disease. There are some reports indicating that β-blockers may be beneficial in preventing osteoporosis and fractures [Rejnmark et al., 2004, 2006; Pasco et al., 2005; Reid et al., 2005a, b; Turker et al., 2006; Bonnet et al., 2007] by counteracting the hypothalamic sympathetic outflow to bone [Karsenty, 2001; Takeda et al., 2002], but their role is still unclear. Because anti-TGF-β therapies have been shown to reverse lung disease and improve bone mass and quality, it may provide an opportunity to intervene in pathogenesis of bone disease of MFS. Nevertheless, based on existing literature, there is not enough evidence to support the use of bone anabolic or anti-resorptive therapy at this time. A larger longitudinal study is necessary to evaluate the natural history of bone disease in MFS and potential therapies.
